# Anti-Inflammatory Effects of HDL in Mice With Rheumatoid Arthritis Induced by Collagen

**DOI:** 10.3389/fimmu.2018.01013

**Published:** 2018-05-11

**Authors:** Yunlong Wang, Shulai Lu, Guoqing Zhang, Shaofeng Wu, Ying Yan, Qingzhe Dong, Bin Liu

**Affiliations:** ^1^Biological Specimen Bank, Affiliated Hospital of Qingdao University, Qingdao, China; ^2^Stomatological Department, Qingdao Municipal Hospital, Qingdao, China; ^3^Affiliated Hospital of Qingdao University, Qingdao, China; ^4^Department of Biochemistry, Medical College, Qingdao University, Qingdao, China; ^5^Department of Pharmacology, Qingdao University, Qingdao, China; ^6^Department of Rheumatology, Affiliated Hospital of Qingdao University, Qingdao, China

**Keywords:** high-density lipoprotein, rheumatoid arthritis, inflammation, inflammatory cytokines, collagen

## Abstract

**Objective:**

To investigate the anti-inflammatory effects of high-density lipoprotein (HDL) in mice with rheumatoid arthritis (RA) induced by collagen.

**Methods:**

Male DBA/1 mice (8-week-old) were divided into three groups: control (treated with saline), collagen-induced arthritis (CIA), and CIA + HDL. CIA was induced with bovine type II collagen, and after the injection of bovine type II collagen, the CIA + HDL group received an injection of HDL on day 28 followed by HDL injections four times every 3 days. Mice were weighed, the paws were scored, and paw thickness was measured beginning on day 21. Additionally, the levels of tumor necrosis factor-alpha (TNF-α) and IL-6 were measured by ELISA kits, tissue sections of paws were stained with hematoxylin and eosin, and the inflammatory signaling pathway was analyzed by western blotting.

**Results:**

We found that the production of pro-inflammatory cytokines TNF-α and IL-6 in mice which received HDL decreased 45.14 and 35.02%, respectively. And we also found that HDL could significantly decrease the level of anti-type-II-collagen IgG2a and inhibit the neutrophil infiltration and cell proliferation and protect the ankle joint from type II collage-induced injury. Western blot analysis indicated that HDL could also inhibit the activation of the NF-κB, MAPK, and ERK signaling pathways in RA mice.

**Conclusion:**

HDL can inhibit the inflammation induced by bovine type II collagen and the development of RA.

## Introduction

In 1993, Levine et al. reported that the increase in the plasma levels of tumor necrosis factor-alpha (TNF-α), as well as the mortality rates caused by endotoxin (lipopolysaccharide), were reduced in transgenic mice in which plasma levels of high-density lipoproteins (HDLs) were twofold higher than normal (1). Additionally, HDL has been shown to exert anti-inflammatory effects both *in vitro* and *in vivo*. HDL can downregulate the expression of toll-like receptor-induced pro-inflammatory cytokines, such as TNF-α, IL-6, and endothelial cell vascular cell adhesion molecule 1, and can increase endothelial nitric oxide synthase production. HDL has also been shown to reduce monocyte CD11b expression and migration along a monocyte chemotactic protein-1 gradient. These potent anti-inflammatory properties of HDL may be critical for protection against other inflammatory diseases (2, 3).

Rheumatoid arthritis (RA) is a systemic inflammatory autoimmune disease that causes joint destruction, as evidenced by radiological findings, such as joint space narrowing and bone erosion, which are related to functional disability. Both B cells and T cells aggregate in the synovium of inflamed joints and mediate the pathogenesis of RA. Studies have shown that an increase of proinflammatory cytokines, such as IL-6 and TNF-α, is critically associated with the histological characteristics of arthritis (4–6). Humans with autoimmune disorders such as RA and mouse models of this disorders are associated with increased atherosclerosis (7), HDL is powerful antiatherogenic factor, and it also influence the migration of dendritic cells (7, 8). In addition, HDL had demonstrated to exert a direct immune-modulatory function on T-lymphocytes *in vitro* (9). Based on these functions of HDL on inflammation and immune, HDL may have a positive effect on RA. To our knowledge, there are no clinical studies showing the direct relationship between HDL and RA. It remains unclear about the effect of HDL on RA. Thus, in this study, we used an RA mouse model to investigate the anti-inflammatory effect of HDL on collagen-induced arthritis (CIA) in mice.

## Materials and Methods

### Reagents and Antibodies

Immunization-grade bovine type II collagen solution was purchased from Chondrex, Inc. Freund’s complete adjuvant (FCA) was purchased from Sigma. ELISA kits were purchased from Yuan Ye Technologies. Human HDL came from ProSpec-Tany Technogene Ltd. The p44/42 MAPK (Erk1/2), P-p44/42 MAPK (Erk1/2), P-IκBα, p38, P-p38, and NF-κB p65 primary antibodies were purchased from CST. The GAPDH secondary antibody was purchased from Epitomics, and the lamin B secondary antibody was from Santa Cruz.

### Construction of the CIA Model and Treatment With HDL

Eight-week-old male DBA/1 mice were purchased from the Beijing Vital River Laboratory Animal Technology Co. and maintained under specific bacteria-free conditions. All experiments were approved by the Animal Use Committee of the affiliated hospital of Qingdao University. The mice were divided into three groups: control (treated with saline), CIA, and CIA + HDL. The CIA and CIA + HDL groups were induced according to a previous report (10). On day 0, DBA/1 mice were immunized with bovine type II collagen in an emulsion with FCA. For boosted immunization, DBA/1 mice were prepared and re-injected on day 21 with bovine type II collagen in an emulsion with FCA. The CIA + HDL group also began to receive HDL (10 mg/kg body weight) by tail vein on day 28; an additional injection was given every 3 days for a total of five doses. Mice were weighed, the paws were scored, and paw thickness was measured beginning on day 21. Scoring for the severity of arthritis was measured on a scale of 0–3 for each paw, and scores corresponded to the following characteristics: 0, normal, no inflammation or redness; 1, redness and swelling in one digit; 2, redness and swelling in more than one digit, or redness and swelling in one digit, ankle, wrist joint; and 3, redness and swelling in all digits and joints.

### Measurement of Cytokines and Anti-Collagen II Antibody

Blood was collected from the retro-orbital sinus of the mice at the end of the study. TNF-α, IL-6 levels were measured using ELISA kits according to the manufacturer’s instructions.

The serum level of anti-collagen-II antibody IgG2a was determined by an ELISA method as described (11). Briefly, 96-well microtiter plates were coated with collagen II overnight and washed with PBS, and then 100 µl serum dilutions in PBS were applied to the wells, washed with PBS, and then the HRP-conjugated goat anti-mouse IgG antibody was applied. The plates were read with an ELISA reader at 450 nm, and the values were represented in arbitrary OD units.

### Hematoxylin and Eosin (H&E) Staining

Mice were sacrificed at day 60, and the paws were fixed in 10% neutral buffered formalin, decalcified by immersion in 10% EDTA solution for 1 month, and embedded in paraffin. Tissue sections were stained with H&E. Images were captured with a Nikon TE2000-S (Nikon, Tokyo, Japan) and analyzed.

### Western Blotting

Mice were sacrificed at day 60, the ankle joint was separated, proteins were extracted from the tissue, and protein concentration was measured using a BCA assay kit. Equal amounts of protein per sample were loaded on 12% pre-cast SDS-PAGE gels with MOPs buffer, and following protein separation, proteins were transferred to PVDF membranes. Membranes were incubated for 2 h at room temperature with 5% BSA in Tris buffer and overnight at 4°C with specific primary antibodies. Next, membranes were washed with TBST, incubated with secondary antibodies for 1 h, and rewashed more than three times. Finally, immune reactivities were visualized and scanned into a computer. Individual bands were analyzed with Image J software.

### Statistical Analysis

Data were analyzed using one-way analysis of variance followed by least significant difference determination using SPSS 22.0 software for Windows (SPSS Inc., USA). A value of *p* < 0.05 was considered to be statistically significant.

## Results

### HDL Can Inhibit CIA Development

**Figure 1:**
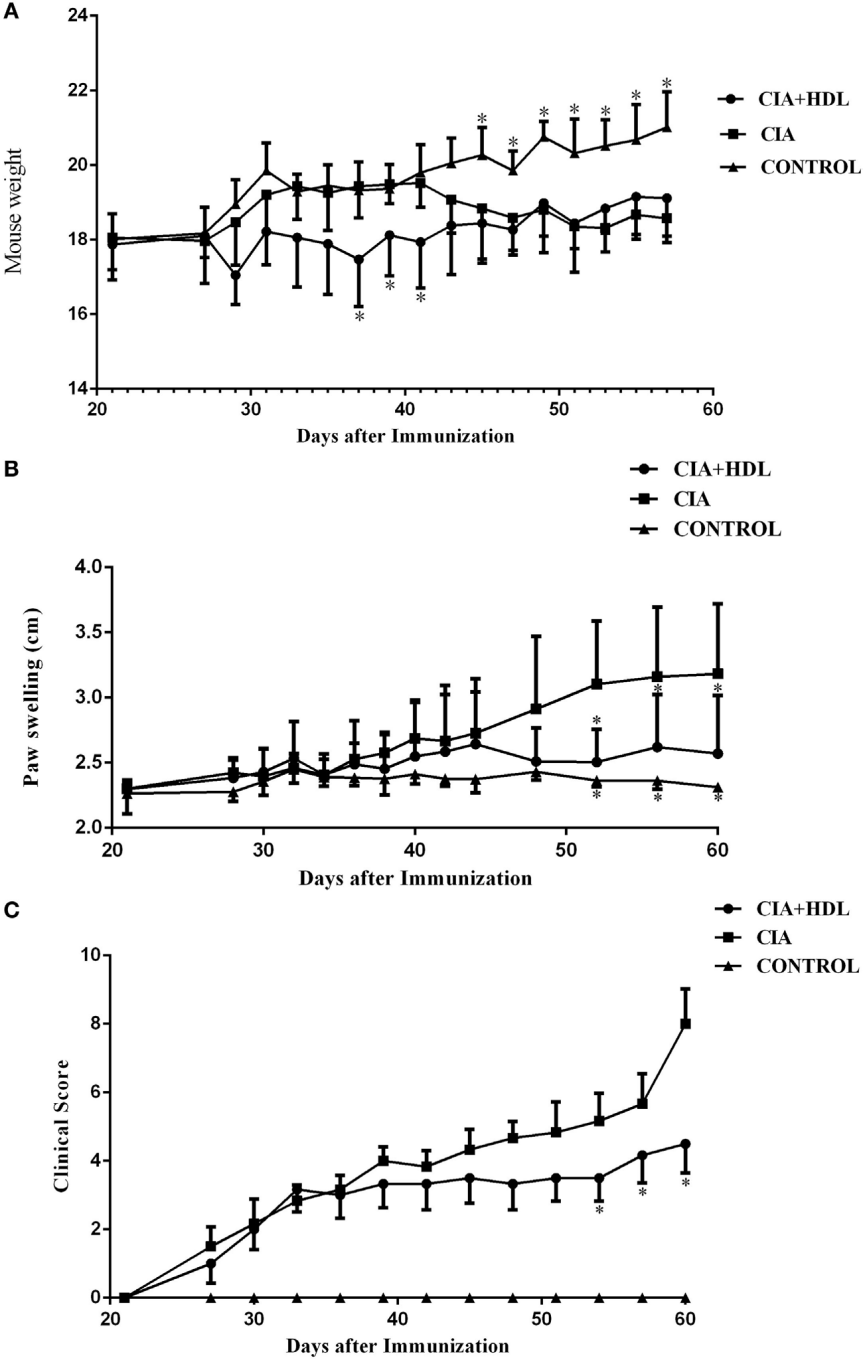
**(A)** Change of body weight in mice; **(B)** degree of paw swelling; **(C)** clinical score of arthritis. The body weight of the collagen-induced arthritis (CIA) + high-density lipoprotein (HDL) group decreased after HDL injection while later increased gradually to a value higher than the body weight of CIA group after day 49. The paw swelling of the mice in CIA + HDL group began to decrease after day 44; however, the CIA group was still increase. Compare with CIA group, the severity of arthritis score tended to decline after the injection of HDL. *p < 0.05 vs. CIA.

Changes in the body weights of the mice in each group showed that the CIA model group and the CIA + HDL treatment group began to decrease on day 28. In Figure 1A, we illustrate that the body weight of the CIA + HDL group decreased after HDL injection and later increased gradually to a value higher than the body weight of the CIA group after day 49. The weight of CIA group increased gradually after stimulation and then began to decrease 40 days later. As seen in Figure 1B, the paw thickness of the mice in CIA group increased gradually, while the paw thickness of the mice in CIA + HDL group began to decrease after day 44. The severity of arthritis score is an important indicator reflective of CIA level. The severity score tended to decline after the injection of HDL in the CIA + HDL group, as shown in Figure 1C. These results indicate that inflammation was restrained by HDL. The degree of swelling in the affected areas reflected the severity of inflammation.

### HDL Decreases the Production of the Pro-Inflammatory Cytokines IL-6 and TNF-α in Serum

**Figure 2:**
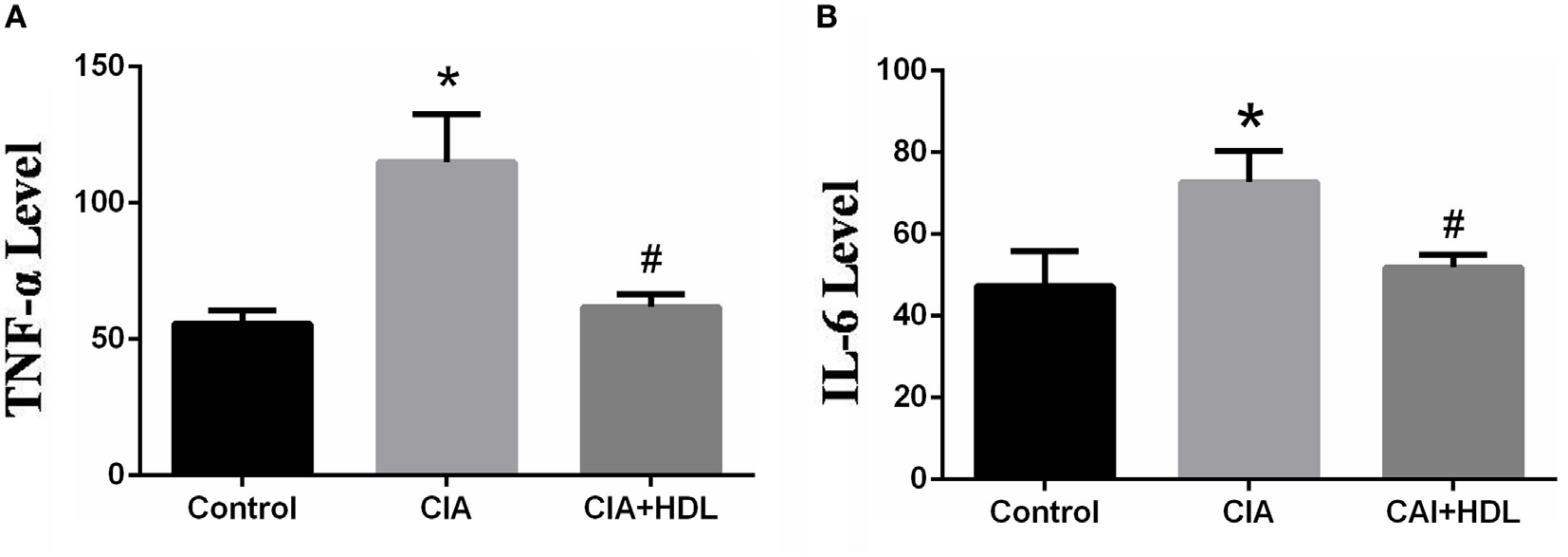
**(A)** The expression of tumor necrosis factor-alpha (TNF-α) in serum; **(B)** the expression of IL-6 in serum. *p < 0.05 vs. control; ^#^*p* < 0.05 vs. collagen-induced arthritis (CIA). The results indicated that the production of pro-inflammatory cytokines TNF-α and IL-6 in mice which received high-density lipoprotein (HDL) decreased 45.14 and 35.02%, respectively.

Inflammatory factors TNF-α and IL-6 reflect the arthritic development stage of CIA mice. As depicted in Figure 2A, the expression level of TNF-α in serum was markedly increased in CIA group mice compared with control group mice (control: 55.69 ± 8.372, CIA:112.6 ± 9.373). Additionally, the expression level of TNF-α in the CIA + HDL group was clearly lower than that of the CIA group (CIA: 112.6 ± 9.373, CIA + HDL: 61.77 ± 1.001), while there were no obvious differences from the control group. In Figure 2B, the expression level of IL-6 in serum was significantly increased in CIA group mice compared with control group mice (control: 47.26 ± 7.563, CIA: 72.74 ± 8.803), and the expression level of IL-6 in the CIA + HDL group was clearly lower than that of the CIA group (CIA: 72.74 ± 8.803, CIA + HDL: 51.86 + 1.592).

### HDL Decrease the Level of Anti-Collagen-II IgG2a

**Figure 3:**
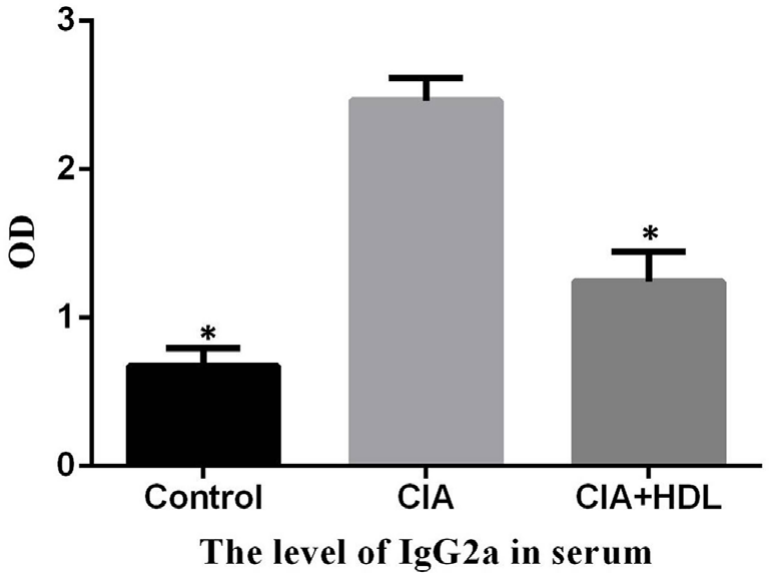
The serum level of anti-collagen-II IgG2a. Compared with the collagen-induced arthritis (CIA) group, the level of IgG2a in CIA + high-density lipoprotein (HDL) group were significantly lower than that of CIA (*p < 0.05 vs. CIA).

To determine the effect of HDL on anti-collagen-II antibody, the level of anti-collagen-II IgG2a in serum was measured by ELISA. And the result showed that the level of IgG2a in CIA + HDL group were significantly lower than that of CIA (*p* < 0.05) (Figure 3).

### HE Staining

**Figure 4:**
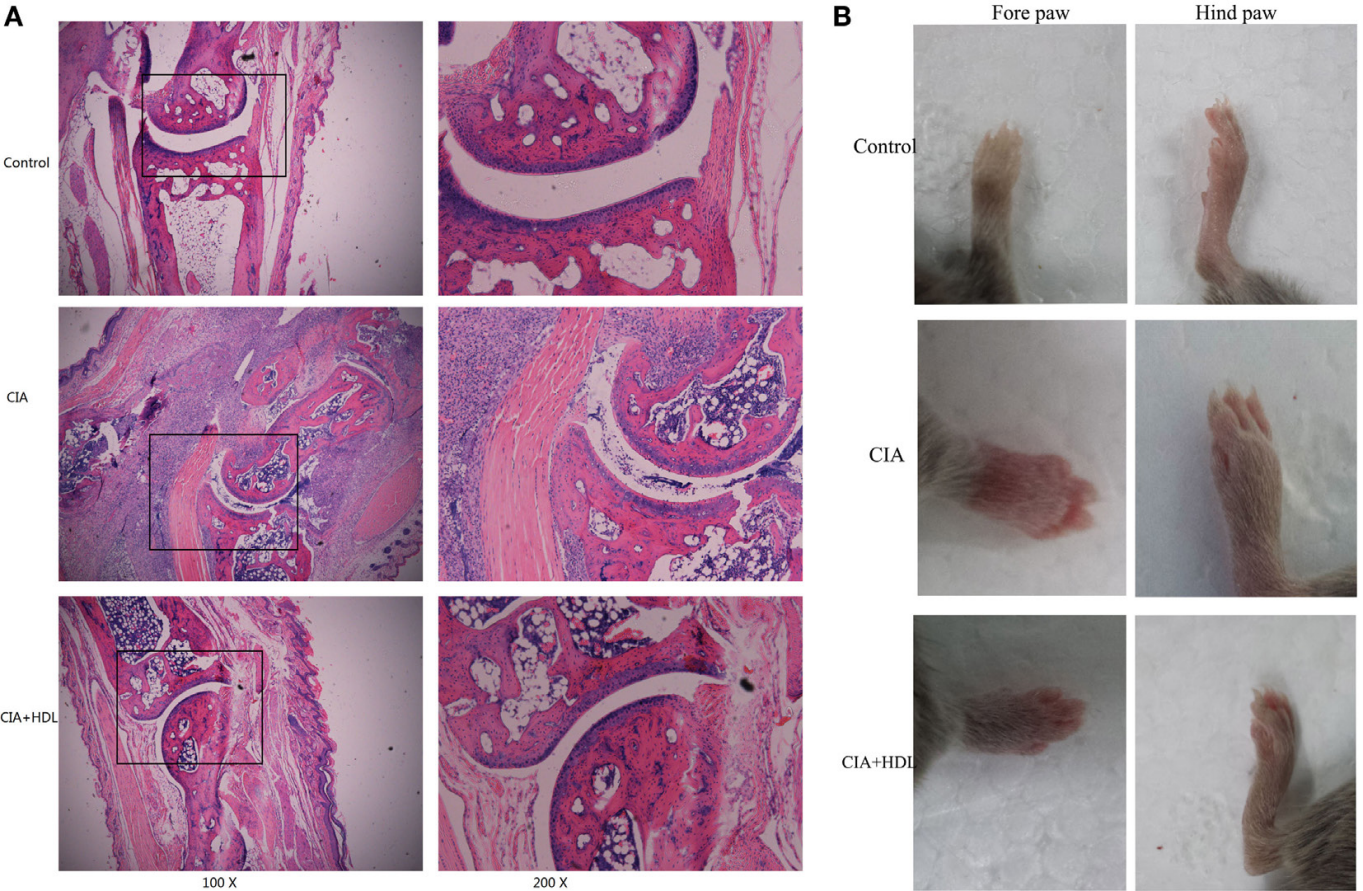
Pathological analysis of mice ankle joints. **(A)** Hematoxylin and eosin staining of the ankle joint; **(B)** the forepaw and hind paw of the mice. Compared with the control group, the joints of the mice in the collagen-induced arthritis (CIA) group exhibited significant pathological changes, while after treatment with high-density lipoprotein (HDL), the pathological changes were significantly decreased **(A)**. We could see in **(B)** that treatment with HDL significantly reduced paw swelling.

We performed HE staining to further assess ankle joint inflammation induced by collagen. Compared with the control group, the joints of the mice in the CIA group exhibited significant pathological changes, including neutrophil infiltration, cell proliferation, and disorderly cell arrangements. After treatment with HDL, we observed that although there was limited cell proliferation and inflammatory cell infiltration, the pathological changes were significantly reduced in comparison with untreated mice (Figure 4A). As Figure 4B illustrates, we observed that compared with the control group, the paws of the CIA group were obviously swollen and red, but treatment with HDL significantly reduced paw swelling.

### Western Blot Analysis

**Figure 5:**
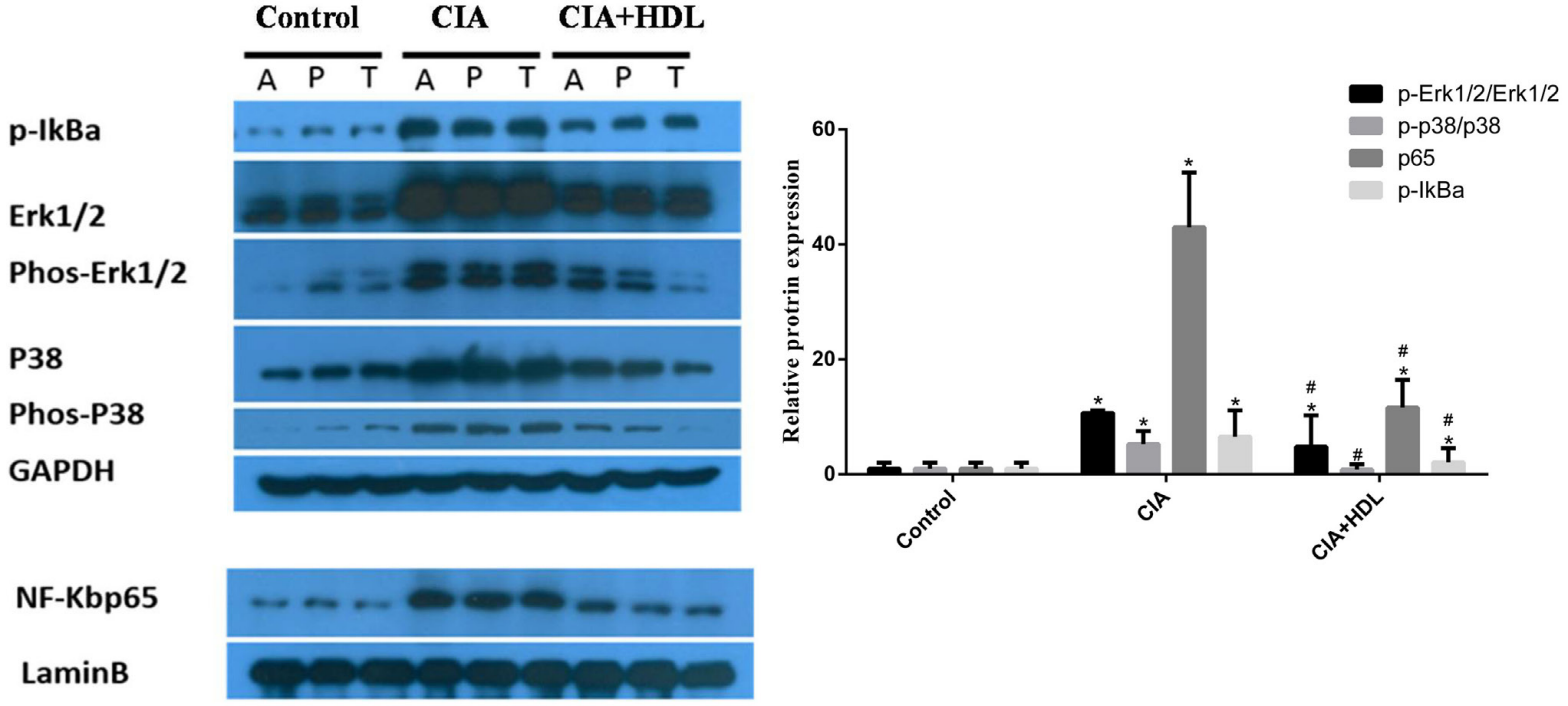
High-density lipoprotein (HDL) inhibits the inflammatory pathway induced by collagen. Compared with the collagen-induced arthritis (CIA) group, we found that HDL could inhibit the activation of the ERK, P38, and NF-kB inflammatory pathway based on their expression levels. *^#^p* < 0.05 vs. CIA group; *p < 0.05 vs. control group.

To investigate the potential mechanisms of the effects of HDL on RA induced by collagen, we collected the sample from joint tissue, and then examined the expression of proteins in inflammatory signaling pathways. As shown in Figure 5, compared with the control group, the expression of p65 in CIA group mice was high (42.97 ± 9.51) but decreased sharply after treatment with HDL (11.58 ± 4.82). Similar to p65, the expression of p-IkBa also decreased after the treatment with HDL (CIA: 6.53 ± 4.57; CIA + HDL: 2.1 ± 2.45). We also observed that after treatment with HDL, the expression of p-Erk1/2/Erk1/2 sharply decreased from 10.68 ± 0.45 to 4.81 ± 5.45, and p-p38/p38 expression also decreased from 5.24 ± 2.26 to 0.81 ± 0.93.

## Discussion

Cytokines and other inflammatory molecules can be detected in the joints of RA patients. IL-1, IL-6, and other cytokines have been proposed to explain the mechanism of inflammation (12, 13). Specifically, TNF-α has been shown to play a key role in the onset and progression of RA. TNF-α is an important cytokine and a vital mediator of inflammation, infection, and autoimmune disease. Prior studies have demonstrated that TNF-α-neutralizing antibody can decrease secretion of IL-1, IL-8, granulocyte colony stimulating factor and the other cytokines from RA synovial cells. Anti-TNF-α-antibody can also alleviate collagen-induced inflammation of RA in mice (14), and *in vivo* data have confirmed earlier *in vitro* findings that show regulation of many cytokines such as IL-6, IL-8 expression in RA synovial tissue by neutralizing TNF-α activity (15). Thus, the inhibition of TNF-α has been demonstrated to be an efficient strategy for the treatment of RA. In our previous study, we found that HDL can regulate TNF-α expression levels (16). Based on these data, we hypothesized that HDL could inhibit RA development. In this study, we used a CIA mouse model that displayed many of the pathological features of human RA. We found that HDL suppressed CIA development and protected against morphological and anatomical joint destruction. Additionally, HDL treatment inhibited the expression of TNF-α in CIA mice, which is in accordance with our previous study (16). We also investigated the expression level of IL-6, which also plays a crucial role in the development and treatment of RA (17, 18). Increased level of IL-6 in CIA mice was also downregulated by HDL. These results support the reasonable conclusion that HDL decreases the expression of the pro-inflammatory cytokines TNF-α and IL-6 (Figure 2) in RA mice.

Additionally, we explored the effect of HDL on the inflammatory signaling pathway in CIA mice. Western blotting results showed that expression levels of ERK1/2 and p38MAPK in the MAPK pathway and p-IκB and p65 in the NF-κB pathway were reduced after treatment with HDL. Thus, HDL can inhibit the activity of inflammatory signaling pathways in RA mice, which is consistent with previous studies (19–21).

In addition, besides the reduced TNF-α expression and activation of inflammatory signaling pathway, S1P may also play an important role in therapeutic effects of HDL in RA. ApoM-bound S1P is a key component of HDL and is responsible for several HDL-associated protective functions (22). Many studies had shown that sphingosine-1-phosphate could attenuate organ injury and decrease the ability of the proinflammatory cytokine TNF-α to activate NF-κB (23, 24). In our study, we also found that HDL could inhibit the inflammation including decreased inflammatory factors level and reduced inflammatory cell infiltration, which may partly associated with its key component of S1P. And this hypothesis will be tested in our future experiments.

In conclusion, we analyzed the effect of HDL on CIA in mice. We observed that HDL was associated with reduced inflammatory RA conditions *in vivo*, including decreased expression of inflammatory factors and reduced activity of inflammatory signaling pathways. Thus, HDL has the potential therapeutic effects in RA.

## Ethics Statement

All experiments were approved by the Animal Use Committee of the affiliated hospital of Qingdao University.

## Author Contributions

YW and BL conceived and designed the experiments. YW, SL, GZ, SW, YY, and QD performed the experiments. SL and YW analyzed the data and wrote the paper. YW and BL contributed reagents/materials/analysis tools.

## Conflict of Interest Statement

The authors declare that the research was conducted in the absence of any commercial or financial relationships that could be construed as a potential conflict of interest.
